# Overview of care for head and neck Cancer cases in Brazilian Cancer Centers during the COVID-19 pandemic^☆^^☆☆^

**DOI:** 10.1016/j.bjorl.2025.101638

**Published:** 2025-06-04

**Authors:** Sergio Gonçalves, Marco Aurélio Vamondes Kulcsar, Leandro Luongo de Matos, José Guilherme Vartanian, Genival Barbosa de Carvalho, Fernando Luiz Dias, Terence Pires de Farias, Emilio Tosto Neto, Carlos Roberto dos Santos, Lucas Gomes Silva, Afonso do Carmo Javaroni, Gyl Henrique Albrecht Ramos, Beatriz Godoy Cavalheiro, José Carlos de Oliveira, Rafael de Cicco, Luiz Eduardo Barbalho de Mello, Bartolomeu Cavalcanti de Melo Junior, Luiz Roberto Medina dos Santos, Rafael Nunes Goulart, Luiz Paulo Kowalski

**Affiliations:** a Instituto do Câncer do Estado de São Paulo (ICESP), Faculdade de Medicina, Universidade de São Paulo (USP), São Paulo, SP, Brazil; b A.C. Camargo Cancer Center, São Paulo, SP, Brazil; c Instituto Nacional de Câncer (INCA), Rio de Janeiro, RJ, Brazil; d Hospital do Câncer de Barretos, Barretos, SP, Brazil; e Hospital Aristides Maltez, Salvador, BA, Brazil; f Hospital Amaral Carvalho, Jaú, SP, Brazil; g Hospital Erasto Gaertner, Curitiba, PR, Brazil; h Instituto Brasileiro de Controle do Câncer (IBCC), São Paulo, SP, Brazil; i Hospital de Câncer Araújo Jorge, Goiânia, GO, Brazil; j Instituto Arnaldo Vieira de Carvalho, São Paulo, SP, Brazil; k Hospital Dr. Luiz Antônio, Liga Norte-Rio-Grandense contra o Câncer, Natal, RN, Brazil; l Hospital de Câncer de Pernambuco, Recife, PE, Brazil; m Centro de Pesquisas Oncológicas (CEPON), Florianópolis, SC, Brazil

**Keywords:** COVID-19, Head and neck neoplasms, Surgery, Attention

## Abstract

**Objective:**

To assess the impact of the COVID-19 pandemic on Brazilian Head and Neck (H&N) oncology reference services.

**Methods:**

This cross-sectional study evaluated data on the number of outpatient consultations, operations and infected physicians collected in 13 Brazilian Cancer Centers.

**Results:**

10% of the H&N surgeons had COVID-19 infection. Significant reduction in the average number of new treated cases (39.9%), consultations (63.1%) and surgeries (35.1%) was observed in April 2020 compared with those of the same period in 2019.

**Conclusion:**

There was significant impairment in the delivery of care to H&N cancer patients during a month of pandemic. The observed reduction is expected to persist in the coming months and will require institutional efforts to adequately meet the demand imposed during the pandemic period.

**Level of evidence:**

Level IV.

## Highlights

• The rate of Covid infection among staff members where the same among all centers.
• The number of appointments decreased.
• The number of surgeries decreased.

## Introduction

The first cases of the 2019 novel Coronavirus Disease (COVID-19), a serious highly transmissible systemic disease caused by a new coronavirus (SARS-CoV-2), were recorded in Wuhan, Hubei province, in China, in December 2019. The World Health Organization (WHO) declared this new disease to be a public health emergency of international concern on 30th January 2020 (Sohrabi et al.),^1^ generating an alert in the medical-scientific community worldwide. With the great advance of cases in several countries, the WHO characterized the situation as a pandemic on 11th March 2020. According to the Johns Hopkins Dashboard,^2^ 5,324,240 cases and 338,286 related deaths were registered in the world until 24 May 2020. In Brazil, 347,398 cases and 22,013 deaths were officially registered until the same date.

While attention to this new threat to public health is prioritized, all other diseases continue to occur. Some of them have high prevalence and severity rates, such as chronic non-communicable diseases, including cancer. In five months, it is estimated that 7,616,232 new cases of cancer and 3,981,261 related deaths were recorded worldwide (IARC), and it is believed that the impact of the COVID-19 pandemic on access to the diagnostic and therapeutic means of that disease will increase its lethality by up to 20% specifically in the UK (Lai et al.).^3^ Head and neck (H&N) cancer account for 6 %–8 % of the cases of malignant neoplasms and one of its prognostic factors is the delay in Time to Treatment Initiation (TTI) from the diagnosis (Graboyles et al.).^4^

In Brazil, data from the National Cancer Institute (INCA) showed that there were 6,295 and 3,899 deaths from oral cavity and larynx cancers in 2017, respectively. It is estimated that there will be 15,190 and 7,650 new cases of malignant neoplasm of the oral cavity and larynx, respectively, in the country in 2020 (INCA).^5^ Most of these patients have their tumors diagnosed in advanced stages, significantly increasing treatment costs for the Brazilian Unified Health System (SUS) (Medici).^6^

Cancer patients were at higher risk of developing serious events when affected by COVID-19 (admission to Intensive Care Unit – ICU, invasive ventilation, or death) compared with non-cancer patients (39% vs. 8%) (Liang et al.).^7^ Public health measures implemented to control COVID-19 recommended the reduction of elective consultations and hospitalizations in several countries, hindering the access of cancer patients to diagnostic and therapeutic procedures (Kowalski et al.).^8^ For this reason, recommendations have been revised with the aim of overcoming current challenges, and thus protecting patients from the risks of cancer progression resulting from delays in TTI (Chaves et al.).^9^ The consequences of the COVID-19 pandemic are catastrophic for a chronically overburdened and underfunded health system as that of Brazil. In addition to the need to maintain social distancing and isolation for a long time, the growing economic and transport difficulties undermine the mobility of patients with chronic diseases. The situation is worsening with the occupation of hospital beds by patients with COVID-19 and the depletion of material and human resources. In this scenario, there has been a remarkable and extremely worrying reduction in the admission of new patients to oncology services.

Cases accumulate in the community while the disease continues its inexorable progression. The prospects are bleak, as in a few months the cancer centers will have to admit an accumulated number of patients with more advanced disease stages. Planning to meet the worsening demand of post-pandemic cancer care needs to follow ethical and scientific criteria based on unequivocal evidence and rely on adequate material and human resources.

This study aims to provide an overview of H&N cancer patients in Brazilian reference cancer centers during the COVID-19 pandemic and analyze how it affected assisting physicians and medical residents in H&N Surgery services.

## Methods

This is a cross-sectional study whose data were collected through voluntarily completed electronic surveys. Data were collected from 13 Brazilian cancer treatment centers (High Complexity Assistance Units – UNACON – and High Complexity Assistance Centers in Oncology – CACON) specialized in H&N Surgery aiming to assess the use of their operational capacity (number of specialists and monthly consultations and surgeries between April 2019 and April 2020 – between the 6th and 9th weeks after registration of the first case of COVID-19 in Brazil). All data were secondary and have been compiled guaranteeing participant confidentiality. The following number of services were surveyed in the respective Brazilian regions: Southeast, 7 (53.8%); Northeast 3 (23.1%); South 2 (15.4%); Midwest 1 (7.7%). Eleven (84.6%) centers are located in state capitals where occurrence of COVID-19 is higher and involve regions that comprise 91.6% of the Brazilian population (IBGE, 2010^10^ and 95.7% of the specialists in Brazil.

The leaders of each participating service filled in the forms, which were made available electronically for seven days starting on May 2nd 2020 (between the 10th and 11th weeks of the pandemic onset in Brazil), with the requested data. The following information was provided by each center: number of surgeons, number of surgeons with COVID-19, number of medical residents in the specialty, number of medical residents with COVID-19, total number of new cases cared for in the study period, total number of return consultations (patients undergoing cancer treatment or post-therapeutic follow-up) conducted in the study period, and total number of surgeries performed in the study period.

A single researcher compiled all the data and each institution was identified by a letter. The data were analyzed using absolute and relative frequencies for qualitative variables and means and standard deviations for quantitative variables. Considering all services, the grouped values were also analyzed and represented by the respective frequencies, using weighted measures when necessary. No sample calculation was performed considering the exploratory nature of the study.

## Results

Table 1 shows that the number of surgeons ranged from three to 15 per service (mean of 8.6 ± 3.4 surgeons/service). The number of medical residents varied between one and 14 per service (mean of 4.5 ± 4.2 residents/service).

**Table 1:** Characteristics of the of H&N Surgery oncological services analyzed.

State	Region	Number of physicians/surgeons	Number of medical residents
Bahia	Capital	9	4
Goiás	Capital	8	2
Paraná	Capital	3	2
Pernambuco	Capital	12	3
Rio de Janeiro	Capital	8	8
Rio Grande do Norte	Capital	13	1
Santa Catarina	Capital	6	2
São Paulo	Capital	15	14
São Paulo	Capital	10	11
São Paulo	Capital	7	3
São Paulo	Capital	9	1
São Paulo	Countryside	8	6
São Paulo	Countryside	4	1

A similar rate of SARS-CoV-2 infection was observed, with a mean of 10% for both physicians and residents, with predominance of states in the Southeast region (Table 2).

**Table 2:** Rate of specialists and medical residents infected by COVID-19 during the study period.

Service	Infected physicians (N/total)	Infected physicians (%)	Infected residents (N/total)	Infected residents (%)	Total infected (N/total)	Total infected (%)
A	3/15	20%	2/14	14.3%	5/29	17.2%
B	3/8	37.5%	3/8	37.5%	6/16	37.5%
C	0/10	‒	1/11	9.1%	1/21	4.8%
D	0/8	‒	0/6	‒	0/14	‒
E	0/4	‒	0/1	‒	0/5	‒
F	0/6	‒	0/2	‒	0/8	‒
G	0/9	‒	0/4	‒	0/13	‒
H	2/7	28.6%	0/3	‒	2/10	20%
I	0/3	‒	0/2	‒	0/5	‒
J	1/9	11.1%	0/1	‒	1/10	10.0%
K	0/8	‒	0/2	‒	0/10	‒
L	2/13	15.4%	0/1	‒	2/14	14.3%
M	0/12		0/3	‒	0/15	‒
TOTAL	11/112	9.8%	6/58	10.3%	17/170	10%

Significant reduction in the number of new treated cases (39.9%), return consultations (63.1%) and operations performed (35.1%) was observed in April 2020 compared with those of the same period in 2019. Only two services presented a larger number of new cases and three services performed more surgical procedures in April 2020 than in April 2019 (Table 3).

**Table 3:** Reduction in delivery of care (new cases of cancer, outpatient return consultations, and elective surgeries) during the COVID-19 pandemic in April 2020 compared with that of April 2019.

Service	New cases of cancer			Return consultations			Elective surgeries		
	April/19	April/20	% of reduction	April/19	April/20	% of reduction	April/19	April/20	% of reduction
A	146	88	39.7	498	222	55.4	62	30	51.6
B	60	58	3.3	789	301	61.9	78	25	67.9
C	213	41	80.8	2171	812	62.6	160	78	51.3
D	82	41	50.0	1246	379	69.6	80	47	41.3
E	76	59	22.4	640	265	58.6	96	81	15.6
F	48	36	25.0	410	138	66.3	42	29	31.0
G	229	169	26.2	994	455	54.2	146	154	−5.5
H	48	80	−66.7	598	374	37.5	39	43	−10.3
I	17	23	−35.3	435	109	74.9	29	25	13.8
J	12	8	33.3	269	118	56.1	15	17	−13.3
K	117	45	61.5	561	127	77.4	166	88	47.0
L	113	31	72.6	732	212	71.0	200	172	14.0
M	58	54	6.9	1060	329	69.0	163	39	76.1
TOTAL	1,219	733	39.9	10,403	3,841	63.1	1,276	828	35.1

Note: Negative reduction values should be interpreted as an increase in patient attention.

## Discussion

The COVID-19 pandemic has transformed healthcare, especially with respect to cancer treatment. Professional entities and reference centers worldwide have proposed guidelines to consider the risk of contamination of cancer patients, health professionals and health-related professionals aiming to reduce infections and the impact on the treatment of these patients, including suggestions on therapeutic modalities other than surgical (American College of Surgeons).^11^

Patients admitted to the traditional oncology care system significantly interrupt the proposal of social distancing. Visits to the clinic and diagnostic centers, hospitalizations, surgical procedures, and attendance to chemotherapy and radiotherapy sessions result in potential opportunities for viral transmission.

Therefore, cancer treatment services have adopted protective measures aimed at restricting access to individuals infected with SARS-CoV-2, in addition to minimizing the contamination rates of patients and health professionals involved in the treatment (Chaves et al.).^9^ These measures included restricting the movement of people in places of care and wards by limiting the participation of companions in consultations, examinations, and hospitalizations. Face-to-face return and oncological control consultations in stable patients with controlled disease have been replaced in many centers by remote assessments mediated via telemedicine. In other centers, return consultations were delayed indefinitely, reserving face-to-face return consultations only to patients undergoing treatment (American College of Surgeons).^11^

It is worth remembering that, in general, the diagnosis of upper aerodigestive tract cancer depends on direct physical examination, oroscopy and endoscopy, which are processes associated with dispersion of aerosols and high risk of contamination by professionals.

Patients who were already awaiting surgical treatment were subjected to new selection criteria and stricter COVID-19 screening protocols to minimize the risks of transmission during surgery and the associated postoperative complications. Some centers have adopted screening measures for elective surgical patients such as performance of pulmonary Computed Tomography (CT) the day before the operation, in search of parenchymal changes typical of COVID-19, or Real-Time Polymerase Chain Reaction (RT-PCR) 48 h before the procedure, following national and international recommendations (Chaves et al.).^9^

On average, 10% (4.8%–37.5%) of the H&N surgeons working in the services evaluated in this study were contaminated with SARS-CoV-2. High rates of COVID-19 among healthcare professionals have reduced their availability for patient care. A research conducted by the Royal College of Physicians in April 2020 found that approximately 20% of the professionals were withdrawn from their duties at work. The main reasons were the suspicion or confirmation of SARS-CoV-2 infection followed by self-isolation because a family member presented symptoms (Mayor).^12^

There has long been a lack of investments in public health, thus accumulation of cancer consultations and treatments was already experienced even before the current pandemic. In addition, the stricter processes regarding patient transport, medical dressing, circulation control, and availability of personal protective equipment and operating rooms and hospital beds have limited the performance of surgical and diagnostic procedures, as demonstrated in this study by the reduction in the number of cases, return consultations and operations carried out in April 2020 compared with those in same period of the previous year. This reduction has increased the number of patients seeking diagnosis and treatment, lengthened the waiting lines for surgical procedures, impaired the early detection of tumor recurrence or persistence and compromised the prognosis of these patients, thus generating a potentially catastrophic situation.

Small TTI delays (3-months) have a significant impact on survival rates for patients with tumors in advanced stages. Even for cases with a relatively favorable prognosis, a moderate TTI delay (6-months) will result in attributable deaths. Moreover, it will result in more advanced tumors, implying not only decreased survival rates, but also more complex and costly treatments (Sud et al.).^13^

A persistent backlog of cases is expected during the outbreak stipulated by governments. If case accumulation continues at the same pace, following the pattern revealed by analysis of the results, which corresponds to approximately one-third of the volume of care delivery each month, the situation will be unsustainable. As an example, if the absolute number of consultations in the H&N cancer centers analyzed in this study is considered, and if the pandemic lasts for three more months at the same pace, there will be an increase of approximately 1,500 patients to the usual demand and 1,350 new patients awaiting surgical procedures, and these patients will probably be presented with more advanced cancer stages, which will preclude treatment from the oncological point of view.

On the other hand, compared with COVID-19 management, the surgical treatment of cancer is highly impacting in terms of life-years gained by resources spent. Delay in diagnosis and treatment causes an exponential burden of attributable mortality. The COVID-19 pandemic has placed unprecedented pressure on healthcare provision, and it is highly plausible that this strain will continue for up to two years (Sud et al.).^11^ Thus, the situation will require combined efforts to lessen this unprecedented impact on the entire health system. It is up to health professionals and public managers to strategically plan to adequately meet this demand, reorganizing healthcare teams, referral systems, and patient flow to optimize care delivery and resolve cases promptly. These are challenges and learning opportunities.

## Conclusion

This scenario shows significant impairment in the initial care and specialized treatment of patients with H&N cancer during the COVID-19 pandemic period caused by SARS-CoV-2 infection.

The following suggestions, considered effective, are proposed: “shielding” of cancer treatment hospitals for patients with disease suspicion or with COVID-19 through the creation of separate wards so that the flow of patients and companions, which has not yet been tested, be individualized; a system with greater agility in referring these patients to institutions dedicated to the treatment of this disease.

Establishing contamination-free (or at least with maximum control of the flow of patients and health personnel) hospitals and institutes that preserve cancer patients and all health professionals involved, safeguarding the conditions for oncological treatment of patients, seems to be crucial to guarantee the continuity of adequate treatment for cancer patients in Brazil.

## Financial support

None.

## Declaration of Generative AI and AI-assisted technologies in the writing process

The authors declare no conflicts of interest.
